# Rapid reduction of high-level pre-formed donor-specific antibodies after simultaneous liver-kidney transplantation: a report of two cases

**DOI:** 10.1186/s12882-020-01714-y

**Published:** 2020-02-12

**Authors:** Christina Lai, Allyson Newman, Jane Mawson, Frederika Abou-Daher, Narelle Watson, Avik Majumdar, Kate Wyburn, Steve Chadban, David Gracey

**Affiliations:** 1 RPA Transplantation Services, Royal Prince Alfred, Sydney, Australia; 2 Kidney Node, Charles Perkins Centre, University of Sydney, Sydney, Australia; 3 Department of Renal Medicine, Royal Prince Alfred Hospital, Missenden Rd, Camperdown, NSW 2050 Australia; 4 Transplantation and Immunogenetics Service, Australian Red Cross Blood Service, Sydney, Australia; 5 AW Morrow Gastroenterology and Liver Centre, Royal Prince Alfred Hospital, Sydney, Australia; 6 Central Clinical School, Faculty of Medicine, University of Sydney, Sydney, Australia

**Keywords:** Complement-dependent cytotoxic, CDC, Desensitisation, Donor-specific antibodies, DSA, Allograft rejection, Simultaneous liver-kidney transplantation, SLKT, Case report

## Abstract

**Background:**

Kidney transplantation performed in the presence of high-titre donor-specific antibodies (DSA) may result in hyper-acute or accelerated antibody-mediated rejection and rapid allograft loss. Previous studies have shown that this risk may be mitigated with simultaneous liver-kidney transplantation (SLKT); however, the mechanisms are not well defined. Here we report the evolution of pre-formed, high-level DSAs in two highly sensitised SLKT recipients peri-operatively and describe a profound sustained depletion of all DSAs from the time of liver anastomosis with no extra desensitisation therapy required.

**Case presentation:**

Two patients underwent SLKT and received our centre’s standard renal transplant immunosuppression with basiliximab and methylprednisolone for induction therapy and prednisolone, mycophenolate and tacrolimus for maintenance therapy. HLA antibody samples were collected pre-operatively, and immediately post-liver and post-kidney revascularisation, and then regularly in the post-transplant period. Complement Dependant Cytotoxicity (CDC) crossmatches were also performed. Both patients were highly sensitised with a PRA over 97%. One patient had a positive B- and T-cell crossmatch pre-transplant. These positive CDC crossmatches became negative and the level of pre-formed DSAs reduced profoundly and rapidly, within 3 h post-liver revascularisation. The reduction in pre-formed DSAs, regardless of subclass, was seen immediately post-liver revascularisation, before implantation of the renal allografts. No significant reduction in non-donor specific HLA-antibodies was observed. Both patients maintained good graft function with no rejection on kidney allograft protocol biopsies performed at 10-weeks post-transplant.

**Conclusions:**

These cases support the protective immunoregulatory role of the liver in the setting of SLKT, with no extra desensitisation treatment given pre-operatively for these highly sensitised patients.

## Background

In kidney transplantation, the presence of a positive complement-dependent cytotoxic (CDC) crossmatch and a high titre of donor-specific antibodies (DSAs) may be considered a contraindication for transplantation, due to the risk of hyper-acute rejection and subsequent graft loss. In contrast, the presence of preformed DSAs in liver transplant recipients has a much less deleterious clinical effect, with no difference observed in 12-month allograft outcomes in sensitised liver transplant recipients [1]. Simultaneous liver-kidney transplantation (SLKT) may abrogate the risk to the kidney, as illustrated by previous case reports of successful SLKT among highly sensitised patients [2]. Favourable clinical outcomes, even in the presence of unfavourable immunological tests at baseline, means that transplantation may be performed in SLKT patients that would have not routinely be performed in kidney-alone recipients. The mechanisms behind this phenomenon remain unclear. Here we report two highly sensitised SLKT recipients with high-level preformed DSAs who exhibited a rapid and profound reduction in their level of DSAs, commencing immediately from the time of liver revascularisation and prior to the implantation of the kidney.

## Case presentations

Patient 1 was a 60-year-old female who was on the waiting list for SLKT for decompensated non-alcoholic steatohepatitis-related cirrhosis and Stage IV chronic kidney disease, presumed secondary to Type 2 diabetes. She presented to hospital acutely unwell with gallstone pancreatitis complicated by progressive decompensated liver and renal failure. Her pancreatitis resolved after stent placement in pancreatic duct at endoscopic retrograde cholangio-pancreatography (ERCP). She continued to deteriorate clinically, despite the resolution of pancreatitis. Prior to transplant, she had a Model for End-stage Liver Disease (MELD) score of 40 (Sodium 126 mmol/L, Creatinine 492 μmol/L, total Bilirubin 287 μmol/L, INR 2.5) and had commenced acute haemodialysis three times a week. She required multiple blood transfusions prior to transplantation and had a calculated panel reactive antibodies (cPRA) for Class I and II HLA antigens of 99%. She received a combined kidney-liver donor offer from the same donor, against which she had multiple class I and II DSAs with mean fluorescence intensity (MFI) above 20,000, as well as a positive T- and B-cell CDC crossmatch, as shown in Table 1. Given her deteriorating clinical condition, she proceeded to transplantation despite the high immunological risk.

**Table 1:** Pre-transplant immunology profiles of patients 1 and 2

*Patient 1*		
*No. of HLA Mismatches*	4/6	
		^*#*^***C1q assay***
^*#*^*DSA (MFI)*	B60, DQ2, DQA1*05:02, DQ4 (> 20,000)	Positive
	DR8, DR17 (> 10,000)	Negative
	DR52, C*07:02 (> 1500)	Negative
*CDC Crossmatch*		
*T cell*	Positive	
*B cell*	Positive	
*cPRA*	Class I and Class II 99%	
*Patient 2*		
*No. of HLA Mismatches*	6/6	
		^*#*^***C1q assay***
^*#*^*DSA (MFI)*	DR52, DR14 (> 10,000)	Negative
	DR4, DQ5 (> 3000)	Negative
	DQ7 (> 1000)	Positive
*CDC Crossmatch*		
*T cell*	Negative	
*B cell*	Negative	
*cPRA*	Class II only 97%	

(*HLA* Human leukocyte antigen, *DSA* Donor-specific antibody, *MFI* Mean fluorescence intensity, *CDC crossmatch* Complement-dependent cytotoxic crossmatch, *cPRA* calculated panel-reactive antibody)

^#^Sera were tested using LABScreen™ single antigen beads (One Lambda, Canoga Park, CA) with a threshold mean fluorescent intensity ≥500, pre-test dilution was not routinely performed

Patient 2 was a 63-year-old female with a history of autosomal dominant polycystic kidney and liver disease who had been on the deceased donor transplant wait list for 2 years. She was highly sensitised with a cPRA for Class II HLA antigens of 97%; she had a history of 2 previous pregnancies. The T- and B-cell CDC crossmatch was negative; however, she had class II DSAs with MFIs above 10,000, as shown in Table 1. Given her degree of sensitisation, and in the presence of a negative crossmatch, it was deemed reasonable to proceed with this donor to transplantation.

Both recipients underwent SLKT with no pre-transplant conditioning. It was considered clinically unsafe to administer any pre-conditioning therapy to Patient 1 because of her perilous clinical state. In the presence of a negative CDC crossmatch and in view of previous clinical experience at our centre, it was felt that there was no compelling indication for additional desensitisation therapy in Patient 2. Hence, both patients received our centre’s standard renal transplant induction therapy with methylprednisolone and basiliximab, and their maintenance immunosuppression included prednisolone, mycophenolate and tacrolimus with a target trough level of 6-8 ng/ml.

**Fig. 1:**
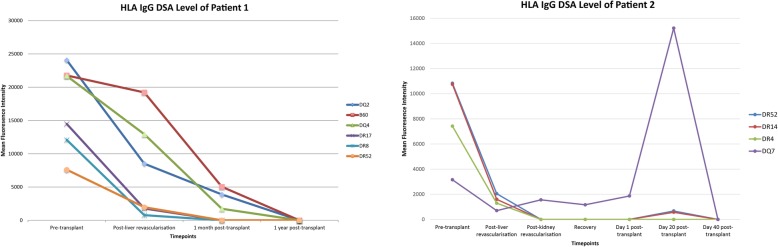
HLA IgG DSA level of Patient 1 and Patient 2

Both patients underwent SLKT as per the centre’s usual practice with no deliberate delay in kidney transplantation. The cold ischaemic times for the liver and kidney allografts were 225 and 407 min respectively for Patient 1; and 348 and 411 min respectively for patient 2. Immediately following liver revascularisation, the level of donor-specific antibodies in both recipients had dropped significantly. Patient 1’s positive crossmatch subsequently became negative within 3 h after liver revascularisation. All DSAs continued to decrease post-transplantation (Fig. 1). This finding was also apparent with the C1q-binding HLA antibody analysis. This reduction was not apparent for non-donor specific pre-formed HLA antibodies.

Patient 2 had a substantial rebound in a single DSA directed against DR7 to an MFI of 15,210 at day 20 post-transplant, whilst maintaining stable renal and liver graft function, as seen in Fig. 1. This was also reflected in the C1q-binding HLA antibody analysis, with MFI of 28,131. In view of these results, and in the absence of any clinical evidence to suggest acute allograft dysfunction caused by this antibody, her Tacrolimus dose was increased to aim for a trough level of 8-10 ng/ml, instead of 6-8 ng/ml. By day 40 post-transplant and after augmented immunosuppression, no DSA was detectable.

**Fig. 2:**
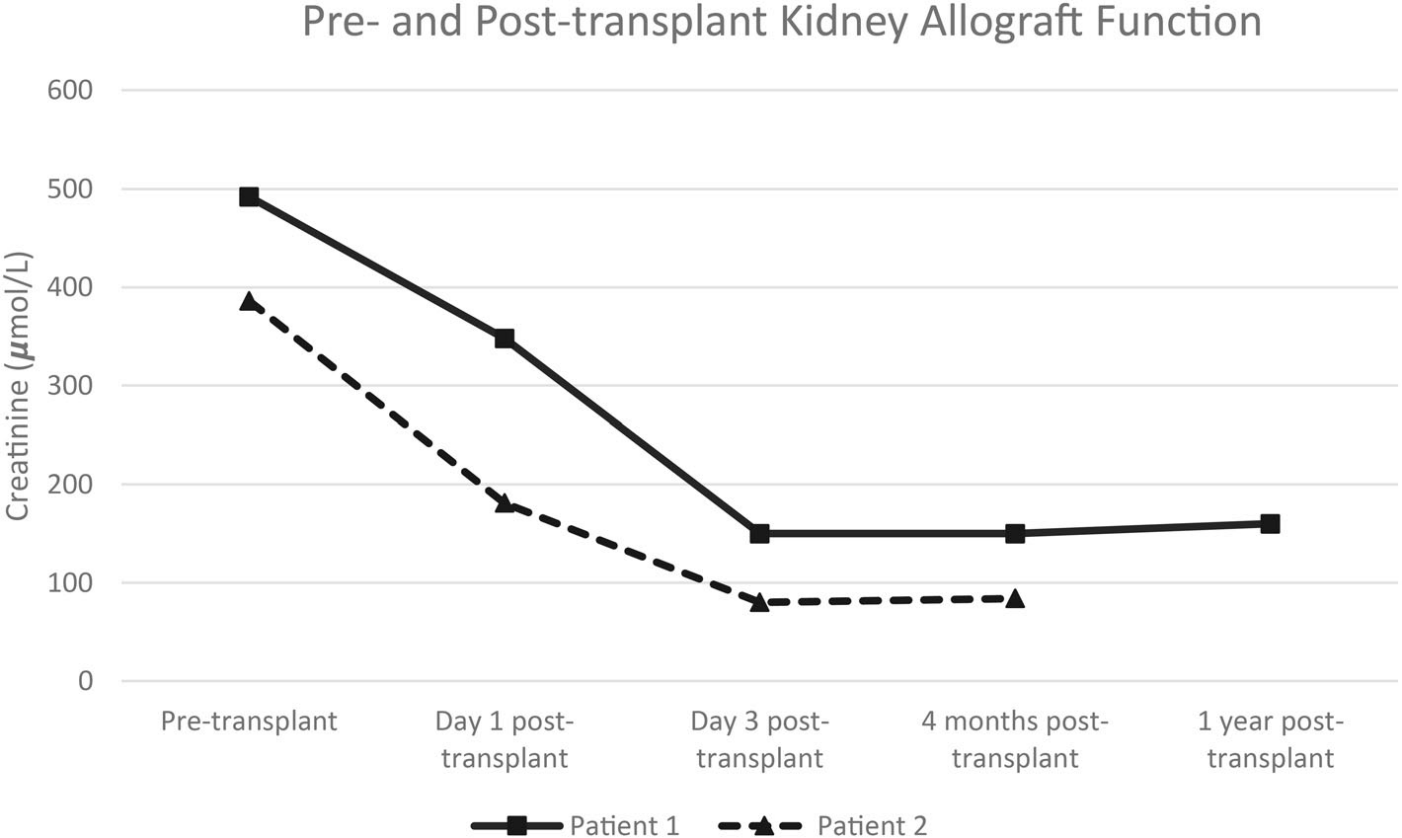
Pre- and Post-transplant Kidney Allograft Function

Both recipients maintained good liver and kidney allograft function, as shown in Fig. 2. As well, no episodes of kidney or liver allograft rejection were observed in either patient. At 10 weeks post-SLKT they both had no rejection on protocol kidney allograft biopsies, with negative C4d immunostaining. Patient 1’s 12-month kidney allograft protocol biopsy was normal with no evidence of rejection (Banff lesion score 0), and no DSA detected in her serum at 1-year post-transplantation. Neither patient has required a liver allograft biopsy, with stable liver allograft function.

## Discussion and conclusions

The liver plays an important role in abrogating the risk of rejection and graft loss in highly sensitised patient undergoing SLKT. Our cases demonstrate a rapid reduction in both Class I and II DSAs post-liver revascularisation, prior to administration of standard induction therapy. Neither patient received extra desensitisation treatment prior to transplantation, and both have had excellent clinical outcomes to date with standard immunosuppression and with no rejection episodes. Although, the follow up of our two cases is relatively short and poorer long-term outcomes have been reported in SLKT patients at later time points [3, 4].

These cases confirm an immunoregulatory role for the liver in protecting the kidney allograft in the setting of SLKT, as has been similarly described in a prospective observational study by O’leary *et. al*. who also noted preformed Class I DSAs, even with MFI values> 10,000, were adequately cleared [5]. However, unlike our cases, the Class II DSAs typically persisted after transplantation. Other studies have also suggested that class II DSAs may persist post-SLKT, predominantly those directed against DQ antigens [4, 6, 7]. It has been hypothesised that the preferential clearance of Class I over Class II DSAs could be attributed to the different expression of Class I and II HLA molecules on the liver allograft [4]. There is a considerably lower density of Class II expression and secretion in the liver parenchyma and vasculature resulting in partial removal of Class II DSA [8]. In patient 2 we observed rebound in a single class II DSA at day 20 post-transplant, which subsequently disappeared with augmented immunosuppression by day 40, with no evidence of rejection of either allograft. To our knowledge, this is the first report that demonstrates the rapid reduction in the MFIs of the DSAs at liver revascularisation, and before the implantation of the kidneys. There was no significant reduction in non-donor specific HLA antibodies demonstrated.

As well as the removal of HLA antibodies, the liver allograft is also capable of rapidly converting a positive T- and B-cell CDC crossmatch to negative. With the use of various pre-transplant desensitisation treatments prior to SLKT, previous case reports have confirmed that this may occur, with the earliest conversion reported at 1 h post-liver revascularisation [9–11]. Patient 1 is the first case report that the authors are aware of where this phenomenon has been demonstrated in the absence of extra desensitisation therapy. In patient 1 the conversion from a positive to negative T- and B-cell CDC crossmatch was observed within 3 h post-liver revascularisation. She had no DSA at 12-month post-transplant with the use of a standard immunosuppression regimen.

Previous reports have illustrated various strategies attempting to abrogate rejection in the setting of a positive crossmatch, including the use of intravenous immunoglobulin, Rituximab and plasmapheresis [12]. Desensitisation; however, did not improve patient allograft outcomes in one Kaplan-Meier analysis [3]. With no additional conditioning or immunosuppression, both of our patients have so far experienced good clinical outcomes with no evidence of rejection to date. It must be borne in mind; however, that poorer long-term outcomes have previously been reported in SLKT patients [5]. These cases may indicate that it is possible to avoid the use of additional immunosuppression therapies at induction whilst still achieving favourable clinical outcomes in the setting of SLKT.

Despite the increasing number of SLKTs performed world-wide [13], there are issues that remain outstanding. Firstly, the optimal induction and maintenance immunosuppressive therapy for these patients is not clear. Our cases suggest that additional desensitisation treatment in these patients may potentially be avoided. Secondly, the precise mechanistic role of the liver in abrogating the clinical effect of the DSAs and attenuating the positive immunological crossmatches in these highly sensitised SLKT patients is not well understood. Traditionally, it was thought the role of the liver is to absorb DSAs. Taner et al. demonstrated that the liver is also capable of shifting the pattern of renal allograft gene expression away from pro-inflammation, thereby preserving renal allograft function [14]. Thirdly, there is a lack of robust long-term clinical data concerning the clinical outcomes of SLKT. Some small studies suggest a higher incidence of transplant rejection, particularly in highly sensitised patients compared to non-sensitised patients, while others have demonstrated lower incidences of both acute and chronic antibody-mediated rejection [3, 5, 15]. Lastly, the significance of delayed implantation of the kidney allograft in highly sensitised patients has not been evaluated. Lunsford et al. and Ekser et al. both found that delayed implantation of kidney allografts was associated with improved patients and renal allografts survival in the SLKT population [16, 17]. However, they have not reported on the degree of sensitisation on their cohort of patients.

These cases demonstrate that high level DSAs and positive T- and B-cell CDC crossmatches should not preclude SLKT in highly sensitised patients, particularly when their risk of remaining un-transplanted is high; however, this report is only of two cases and their long-term clinical outcomes are not yet available. The risk of antibody-mediated rejection amongst these patients remains high, mandating clinical vigilance and close monitoring in the post-transplant period. We undertook regular screening for DSAs in the post-transplant period, initially weekly and then monthly, to monitor for the risk of anti-body mediated rejection, allowing us to augment patient 2’s immunosuppression in response to the rebound seen in their class II DSAs. In selected patients, such as ours, we have observed good outcomes to date with standard immunosuppression and without the use of additional desensitisation prior to SLKT.

## Data Availability

Not applicable.

## Abbreviations

CDC: Complement dependant cytotoxicity
DSA: Donor-specific antibodies
ERCP: Endoscopic retrograde cholangio-pancreatography
HLA: Human leukocyte antigen
INR: International normalised ratio
MELD: Model for end-stage liver disease
MFI: Mean fluorescence intensity
PRA: Panel-reactive antibody
SLKT: Simultaneous liver-kidney transplantation
